# Physiological responses of a halophytic shrub to salt stress by Na_2_SO_4_ and NaCl: oxidative damage and the role of polyphenols in antioxidant protection

**DOI:** 10.1093/aobpla/plu042

**Published:** 2014-07-24

**Authors:** Mariana A. Reginato, Antonella Castagna, Ana Furlán, Stella Castro, Annamaria Ranieri, Virginia Luna

**Affiliations:** 1 Fisiología Vegetal, Físico Químicas y Naturales, Universidad Nacional de Río Cuarto, Río Cuarto, Argentina; 2 Department of Agriculture, Food and Environment, University of Pisa, Via del Borghetto, 80 56124 Pisa, Italy; 3 Biología, Fac. de Cs. Exactas, Físico Químicas y Naturales, Universidad Nacional de Río Cuarto, Río Cuarto, Argentina

**Keywords:** NaCl, Na_2_SO_4_, oxidative damage, pigments, polyphenols, salt stress.

## Abstract

Salt stress conditions lead to increased production of reactive oxygen species (ROS) in plant cells. Halophytes have the ability to reduce these toxic ROS by means of a powerful antioxidant system that includes enzymatic and non-enzymatic components. In this research, we used the halophytic shrub *Prosopis strombulifera* to investigate whether the ability of this species to grow under increasing salt concentrations and mixtures was related to the synthesis of polyphenolic compounds and to the maintenance of leaf pigment contents for an adequate photosynthetic activity. Seedlings of *P. strombulifera* were grown hydroponically in Hoagland's solution, gradually adding Na_2_SO_4_ and NaCl separately or in mixtures until reaching final osmotic potentials of −1, −1.9 and −2.6 MPa. Control plants were allowed to develop in Hoagland's solution without salt. Oxidative damage in tissues was determined by H_2_O_2_ and malondialdehyde content. Leaf pigment analysis was performed by high-performance liquid chromatography with ultraviolet, and total phenols, total flavonoids, total flavan-3-ols, condensed tannins, tartaric acid esters and flavonols were spectrophotometrically assayed. Treatment with Na_2_SO_4_ increased H_2_O_2_ production and lipid peroxidation in tissues and induced a sharp increase in flavonoid compounds (mainly flavan-3-ols) and consequently in the antioxidant activity. Also, Na_2_SO_4_ treatment induced an increased carotenoid/chlorophyll ratio, which may represent a strategy to protect photosystems against photooxidation. NaCl treatment, however, did not affect H_2_O_2_ content, lipid peroxidation, pigments or polyphenols synthesis. The significant accumulation of flavonoids in tissues under Na_2_SO_4_ treatment and their powerful antioxidant activity indicates a role for these compounds in counteracting the oxidative damage induced by severe salt stress, particularly, ionic stress. We demonstrate that ionic interactions between different salts in salinized soils modify the biochemical and morpho-physiological responses of *P. strombulifera* plants to salinity.

## Abstract (precis)

We studied the halophytic shrub *Prosopis* strombulifera to investigate whether the differential ability of this species to grow under increasing salt concentrations and mixtures was related to the synthesis of polyphenolic compounds and to the maintenance of leaf pigment contents for an adequate photosynthetic activity. The significant accumulation of flavonoids in tissues under Na_2_SO_4_ treatment and their powerful antioxidant activity indicates a role for these compounds in counteracting the oxidative damage induced by severe salt stress, particularly, ionic stress. We demonstrate that ionic interactions between different salts in salinized soils modify the biochemical and morpho-physiological responses of *Prosopis strombulifera* plants to salinity.

## Introduction

Increased soil salinity has been a substantial threat to agriculture in some parts of the world for more than 3000 years, and the problem is becoming more widespread through time (Laüchli and Grattan 2007). For this reason, to improve knowledge about the specialized physiology and biochemistry of halophytic plants (known for their exceptional salt tolerance) represents a goal for scientists.

Salt stress leads to increased production of reactive oxygen species (ROS) in plant cells. Reactive oxygen species are extremely reactive and undergo uncontrollable and damaging reactions with cellular components including DNA, lipids and proteins, which can aggravate the detrimental effects of the initial stress and even lead to cell death (Halliwell 2006; Van Breusegem and Dat 2006). Oxidative stress is a central factor in abiotic and biotic stress phenomena, which occurs when there is a serious imbalance in any cell compartment between ROS production and antioxidant defence leading to dramatic physiological challenges (Foyer and Noctor 2003). It was considered that ROS concentration needs to be maintained as low as possible, although this concept is changing because of the multiple functions that are currently being discovered for these molecules (Mittler and Blumwald 2010). Thus, it is important for cells to keep a tight control of ROS concentration, but not to eliminate them completely (Schützendübel and Polle 2002).

Halophytes are known for their ability to withstand unfavourable conditions by quenching these toxic ROS, since they are equipped with a powerful antioxidant system that includes enzymatic and non-enzymatic components. Natural antioxidants occur in all plant organs, and the typical compounds that exhibit antioxidant activities include phenolics, carotenoids and vitamins (Chanwitheesuk *et al.* 2005). Tocopherols and carotenoids protect lipid membranes from oxidative stress because they deactivate singlet oxygen by physical quenching and/or chemical scavenging, and prevent the propagation of lipid peroxidation by reducing fatty acyl peroxy radicals (Polle and Rennenberg 1994; Falk and Munné-Bosch 2010). Enhanced synthesis of particular secondary metabolites under stressful conditions is also believed to protect the cellular structures from oxidative effects (Jaleel *et al.* 2007). Among these compounds, polyphenols (mainly flavonoids) play an important role in the defence against ROS, and their synthesis and accumulation has been proposed to be stimulated in plants under salt stress (Navarro *et al.* 2006; Hernández 2007).

The genus *Prosopis* occurs in arid and semiarid regions, being the major component of such ecosystems in South and North America. Many species within this genus have economic and ecological potential (shade, firewood, food and forage for wildlife and livestock). Some species of *Prosopis*, especially *P. pallida*, *P. juliflora*, *P. tamarugo* and *P. alba* have individuals with rapid growth at seawater salinity or 45 dS m*^−^*^1^ which is nearly 20 times greater than salinities that can be tolerated by annual temperate legumes (Felker 2007).

The spiny shrub *P. strombulifera* (Burkart 1976) ranges from the Arizona desert (USA) to Patagonia (Argentina), and is particularly abundant in high-salinity areas of central Argentina (Córdoba and southwestern San Luis provinces). In these highly salinized soils, proportions of NaCl and Na_2_SO_4_ are generally similar, although in previous studies we found that Na_2_SO_4_ was up to three times more abundant than NaCl in several soil samples (Sosa *et al.* 2005). Similarly, in many countries, NaCl and Na_2_SO_4_ are the most abundant salts in salinized soils (Iqbal 2003; Shi and Sheng 2005; Manivannan *et al.* 2008). For that reason it is important to compare the effects of these two salts on plant growth, in order to understand better the physiological responses of plants in natural environments.

Comparative studies have shown that SO_4_^2−^-based solutions have considerably stronger inhibitory effect on *P. strombulifera* germination than Cl^−^-based solutions at iso-osmotic concentrations (Llanes *et al.* 2005; Sosa *et al.* 2005). Stimulation of shoot growth at *Ψ*_o_ values up to −1.9 MPa (500 mM) NaCl is an interesting halophytic response found in our studies (Reginato *et al.* 2014). Findings in other *Prosopis* species indicate that the NaCl tolerance of *P. strombulifera* exceeds the limits described for most halophytic plants (Catalán *et al.* 1994; Almeida Viégas *et al.* 2004; Felker 2007). However, *P. strombulifera* is much less tolerant to Na_2_SO_4_ than to NaCl. Plants grown in the presence of Na_2_SO_4_ showed immediate and significant reduction of shoot height and leaf number per plant, accompanied by senescence symptoms such as chlorosis, necrosis and leaf abscission (Reginato *et al.* 2014).

*Prosopis strombulifera* plants grown in an increasing gradient of NaCl (250 up to 700 mM) do not develop salt glands in the leaves. Some tissues display vacuolization, and the root system undergoes precocious lignification and/or suberization of endodermal cells, with Casparian strips found much closer to the root tip than in glycophytes. These plants can therefore filter soil solution more efficiently to prevent passage of excess ions to the xylem (Reinoso *et al.* 2004). Na_2_SO_4_ treatment induced structural alterations in cells and tissues, with consequent changes in growth patterns at various levels of organization, and anatomical and histological differences in roots, stems and leaflets, compared with control plants, or plants grown under high NaCl (Reinoso *et al.* 2005).

An interesting feature observed in salt-treated plants in our anatomical studies was the significant accumulation of tannins in all organs, which increased with increasing salt concentration (Reinoso *et al.* 2004, 2005), mainly in Na_2_SO_4_-treated plants. These results demonstrate that plant responses may vary depending on the anion associated with sodium. The aim of the present research was to investigate whether the differential ability of this species to grow under increasing concentrations of Na_2_SO_4_, NaCl and their iso-osmotic mixture was related to oxidative damage leading to an enhanced synthesis of polyphenolic compounds. Maintenance of leaf pigment content for an adequate photosynthetic activity was also investigated.

## Methods

### Plant materials and growth conditions

Pods of *P. strombulifera* were randomly collected from 100 plants within the same population, in the southwestern San Luis province, Argentina. Peeled seeds were scarified with 98 % sulfuric acid for 10 min, washed overnight under running water, rinsed in distilled water and germinated in a Petri dish over two layers of water-saturated filter paper at 37 °C for 24 h. The germinated seedlings with 20-mm-long radicles were grown under hydroponic conditions in black trays (200 seedlings per each tray of 28 × 22 × 10 cm) with 10 % of full-strength Hoagland's solution. The seedlings were self-supported in small holes on the tray cover; the trays were placed in a growth chamber (Conviron E15; Controlled Environments Limited, Manitoba, Canada) under a 16 h light (400 µmol m^−2^ s^−1^) at 28 °C : 8 h dark (20 °C) cycle and 70 % relative humidity. After 1 week, the nutrient solution was changed to 25 % Hoagland's solution (osmotic potential (*Ψ*_o_) = −0.11 MPa). The pH of the medium was 6 in all cases, and, to provide aeration, an aquarium aeration system with a peristaltic pump was used. The complete experiment was performed twice, consecutively (3 trays per treatment each time). Plants were grown hydroponically for 7 weeks (48 days) and allowed to acclimate to the different salt regimes.

### Salt treatment

Salt treatments were initiated after 21 days of seedling growth by adding NaCl and Na_2_SO_4_ pulses of 50 and 38 mmol L^−1^, respectively, for single-salt treatments, or iso-osmotic mixture for two-salt treatment, every 48 h until reaching final *Ψ*_o_ values of −1, −1.9 and −2.6 MPa (verified with a vapour pressure osmometer model 5500; Wescor Inc., Logan, UT, USA), as shown in Table 1. These *Ψ*_o_ values corresponded to plants aged 29, 40 and 48 days, respectively. Control plants remained in 25 % Hoagland's solution (*Ψ*_o_ = −0.11 MPa). At each of these time points, roots and leaves of 30 control plants and 30 salt-treated plants were randomly collected from each tray, frozen with liquid N_2_ and stored at −80 °C for polyphenol and pigment analysis.

**Table 1.** Increasing salt concentrations obtained by sequential addition of pulses every 48 h. Four pulses means 4 × 37.9 mL aliquot of Na_2_SO_4_ L^−1^ Hoagland solution. *Ψ*_o_ values were verified with a vapour pressure osmometer. Bold values indicate the point of sampling.

Salt pulses	mL Na_2_SO_4_ 1 M L^−1^ Hoagland	mL NaCl 1 M L^−1^ Hoagland	Salt mixture mL Na_2_SO_4_ 1 M + mL NaCl 1 M L^−1^ Hoagland	*Ψ*_o_
1° pulse	37.9	50	18.9/25	−0.3
2° pulse	75.8	100	37.9/50	−0.47
3° pulse	113.7	150	56.8/75	−0.65
4° pulse	151.7	200	75.9/100	−0.82
5° pulse (**sampling**)	189.7	**250**	94.8/125	**−1**.**0**
6° pulse	227.5	300	113.8/150	−1.18
7° pulse	265.4	350	132.7/175	−1.35
8° pulse	303.3	400	151.7/200	−1.53
9° pulse	341.2	450	170.6/225	−1.71
10° pulse (**sampling**)	379.2	**500**	189.6/250	**−1**.**9**
11° pulse	417.1	550	208.5/275	−2.06
12° pulse	455.0	600	227.5/300	−2.24
13° pulse	492.9	650	246.4/325	−2.42
Last pulse (**sampling**)	530.8	**700**	265.4/350	**−2**.**6**

### Oxidative damage in tissues

Hydrogen peroxide was measured spectrophotometrically after reaction with KI according to Alexieva *et al.* (2001). The reaction mixture consisted of 0.5 mL of 0.1 % trichloroacetic acid (TCA), leaf extract supernatant, 0.5 mL of 100 mM K-phosphate buffer and 2 mL of reagent (1 M KI w/v in fresh double-distilled water). The blank test consisted of 0.1 % TCA in the absence of leaf extract. The reaction was developed for 1 h in darkness and absorbance was measured at 390 nm. The amount of hydrogen peroxide was calculated using a standard curve prepared with known concentrations of H_2_O_2_.

Lipid peroxidation was determined by estimating the amount of malondialdehyde (MDA), a product of unsaturated fatty acid peroxidation, according to Heath and Packer (1968). Frozen samples (0.15 g) were crushed into a ﬁne powder in a mortar under liquid nitrogen and then mixed with 1.5 mL 20 % TCA. The homogenate was centrifuged at 10 000*g* for 10 min at 4 °C, with the supernatant being used for MDA determination. A mixture of 0.5 mL of extract + 0.5 mL of 0.5 % TBA (thiobarbituric acid) (0.5 g TBA + TCA 20 % to complete to 100 mL) was produced, heated at 95 °C for 25 min, cooled and centrifuged for 10 min. The sample was measured at 532 nm and corrected by non-specific absorption at 600 nm. The concentration of MDA was calculated using an extinction coefficient of 155 mM*^−^*^1^ cm*^−^*^1^.

### Leaf pigment analysis

Pigment concentrations of *P. strombulifera* leaves were determined according to the method reported by Castagna *et al.* (2001). Frozen samples were homogenized in the dark in 100 % HPLC-grade acetone with 1 mM sodium ascorbate then filtered through 0.2 μm filters. The analysis was performed by high-performance liquid chromatography (HPLC) (HPLC P200; Thermo Fisher Scientific, Waltham, MA, USA) using a non-endcapped column (Zorbax ODS column; Chrompack, Raritan, NJ, USA) for pigment separation. Two solvents were used: A (acetonitrile/methanol, 75/25, v/v) and B (methanol/ethylacetate, 68/32, v/v). The separation cycle was 1920 s with a flow rate of 16.67 mm^3^ s^−1^. Pigments were eluted using 100 % A for the first 900 s, followed by a 150-s linear gradient to 100 % B, which continued isocratically until the end of the cycle. The column was allowed to re-equilibrate in 100 % solvent A for 600 s before the next injection. Pigments were detected by their absorbance at 445 nm, and their quantification was realized by the injection of known amounts of pure standard into the HPLC system and the formulation of an equation correlating peak area to pigment concentration. The latter was expressed as nmol g^−1^ DW.

Calculation of the de-epoxidation (DEPS) index was based on the contents of antheraxanthin (A), zeaxanthin (Z) and violaxanthin (V) according to the following equation:

DEPSindex=(0.5A+Z)/(V+A+Z)

### Polyphenol analysis

#### Polyphenols extraction

Dry samples (0.5 g) were ground with liquid N_2_. The plant material was extracted on a magnetic stirrer three times using a total of 90 mL of methanol/water (80 : 20, v/v). The liquid extract was separated by centrifugation (14 000*g*, 15 min) at 4 °C. The final volume was quantified, and the extract, reduced to 16 mL by rotary evaporation, was filtered with a 0.45-μm filter (Minisart) and stored at −80 °C.

#### Phenol compound quantification

Total phenols were determined using the Folin-Ciocalteu method, modified as described by Barbolan *et al.* (2003). Amounts of 1.85 mL of distilled water, 0.125 mL of Folin-Ciocalteu reagent and 0.5 mL of a 20 % sodium carbonate solution were added to 25 μL of liquid extract sample in a test tube, making a final volume of 2.5 mL. The solution was homogenized and left to stand for 30 min, and the absorbance was determined at 750 nm. The total phenols were calculated as milligrams of gallic acid equivalents.

Total flavonoids were determined as described by Kim *et al.* (2003). Assays contained 60 μL of 5 % NaNO_2_, 40 μL of 10 % AlCl_3_ and 400 μL of 1 M NaOH in addition to 100 μL of extract. The solution was diluted with 200 μL of distilled water, and the absorbance was determined at 510 nm. The flavonoid amount was calculated as milligrams of catechin equivalents.

Tartaric acid ester and flavonol contents were determined using the method described by Romani *et al.* (1996). An aliquot of 25 μL of extract was diluted with 225 μL of 10 % ethanol and 250 μL of 0.1 % HCl in 95 % ethanol, and 1 mL of 2 % HCl was then added. The solution was mixed, and the absorbance determined at 320 nm for tartaric acid esters and at 360 nm for flavonols. Tartaric acid ester and flavonol amounts were calculated as milligrams of caffeic acid and quercetin, respectively.

Total flavan-3-ols were determined with *p*-(dimethylamino) cinnamaldehyde (DMACA) reagent, as described by Nigel and Glories (1991). The sample extract (10 μL) was diluted with 90 μL of methanol. Next, 250 μL of HCl (0.24 N in MeOH), 250 μL of DMACA solution (0.2 % in MeOH) and 250 μL of methanol were added. The absorbance was determined at 640 nm, and the total amount of flavan-3-ols was calculated as milligrams of catechin equivalents.

Condensed tannins (proanthocyanidins) were determined in accordance with the method described by Waterman and Mole (1994). Butanol reagent was prepared by mixing 128 mg of FeSO_4_ · 7H_2_O with 5 mL of concentrated HCl and brought to 100 mL with *n*-butanol. An aliquot of 50 μL of extract sample was mixed with 700 μL of butanol reagent and heated at 95 °C in a water bath for 45 min. The sample was cooled, 250 μL of *n*-butanol was added, and the absorbance was measured at 550 nm. The total amount of condensed tannin was calculated as milligrams of cyanidin equivalents. The assays were performed using an Ultro spec 2100 pro UV-visible spectrophotometer (Amersham Biosciences).

#### Antioxidant activity of polyphenolic extracts

2,2′-Azino-bis(3-ethylbenzothiazoline-6-sulfonic acid) (ABTS*) scavenging ability of polyphenolic extracts was determined according to the method described by Re *et al.* (1999). ABTS* was generated by reacting an ABTS aqueous solution (7 mM L^−1^) with K_2_S_2_O_8_ (2.45 mM L^−1^, final concentration) in the dark for 16 h and diluting with ethanol to obtain an absorbance of 0.700 ± 0.020 at 734 nm. About 0.2 mL of appropriate dilution of the extract was added to 1.0 mL ABTS* measuring absorbance at 734 nm after 6 min. The Trolox equivalent antioxidant capacity was subsequently calculated.

### Anatomical analyses

Samples were taken from roots, stems and leaves and placed in FAA (95 % ethanol : glacial acetic acid : 37–40 % formaldehyde : water; 50 : 5 : 10 : 35, v/v) (Reinoso *et al.* 2004, 2005). The dehydration of samples was carried out according to the procedures outlined in Johansen (1940) using graduated solutions of ethanol and xylene. Fully infiltrated tissues were embedded in Histowax (highly purified paraffin wax blended with polymer additives). A series of transverse sections 10-μm-thick were obtained from the sample blocks using a Minot rotary microtome. The sections were triple-stained with haematoxylin, safranin O and fast green FCF as described by Johansen (1940). A coverslip was added to the slides with one or two drops of Depex. A standard Zeiss Model 16 microscope was used to assess the histological preparations and photomicrographs were taken with a Zeiss Axiophot microscope with image capture and digitalization (AxioVision 4.3 with AxioCam HRc camera). To identify tannins, freehand sections were cut from fresh material and treated with ferric chloride (D'Ambrogio and Argüeso 1986).

### Statistical analysis

Data were analysed using InfoStat program (Student Version 2011, Universidad Nacional de Córdoba, Argentina). Two-way general linear model ANOVA was used to determine the effect of each treatment at each osmotic potential. Thus, the factors considered for two-way ANOVA were osmotic potential (*Ψ*_o_) (−1.0, −1.9 or −2.6 MPa) and salt treatment (control, NaCl, Na_2_SO_4_ and salt mixture). Normality was verified with the Shapiro–Wilk test. Homogeneity of variance was verified with the Levenne test. When necessary, data were transformed to meet the assumptions of ANOVA. For cases in which normality and homogeneity of variance were not verified, the non-parametric Kruskall–Wallis test was used. *Post hoc* analysis used the Bonferroni test to determine differences between means. *P* values <0.05 were considered statistically significant.

## Results

### H_2_O_2_ content and lipid peroxidation in tissues induced by salt stress

**Figure 1.**
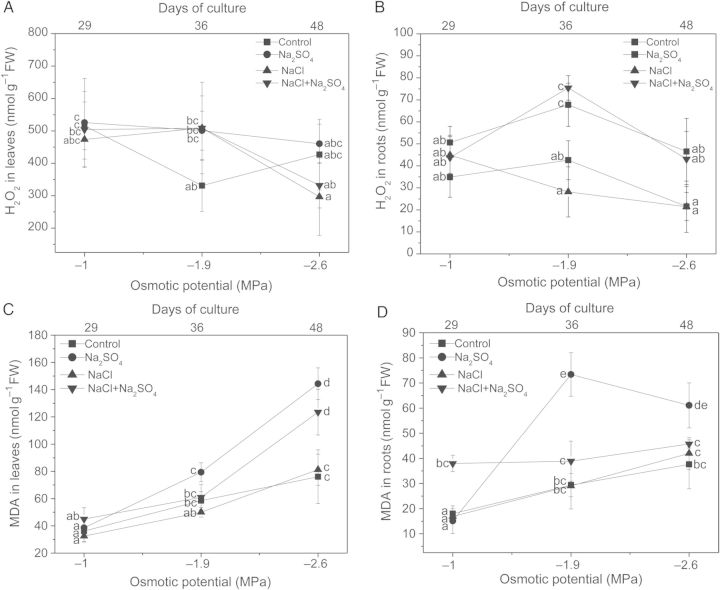
Effect of increasing concentrations of NaCl, Na_2_SO_4_ and their iso-osmotic mixture on H_2_O_2_ content and lipid peroxidation of *P. strombulifera* plants. Hydrogen peroxide content in leaves (A) and roots (B) and MDA content in leaves (C) and roots (D). Salt treatments and osmotic potentials were compared by two-way ANOVA. Means values (±SE) followed by different letters are significantly different at *P* < 0.05 according to Bonferroni test (*n* = 6).

H_2_O_2_ content in leaves remained unchanged in all treatments and was only significantly higher in roots of Na_2_SO_4_ and Na_2_SO_4_ + NaCl-treated plants at −1.9 MPa (Fig. 1A and B).

Salt-induced damage to cellular membranes due to lipid peroxidation was estimated from MDA concentrations. Malondialdehyde was significantly higher in Na_2_SO_4_-treated plants than in NaCl treatments. In leaves, a significant increase of MDA content was observed in Na_2_SO_4_ and Na_2_SO_4_ + NaCl-treated plants at high salinity (−2.6 MPa). Similarly, MDA content increased in Na_2_SO_4_-treated plant roots at moderate and high salinity (−1.9 and −2.6 MPa). NaCl-treated plants showed similar MDA levels to control plants (Fig. 1C and D).

### Leaf pigment content

**Figure 2.**
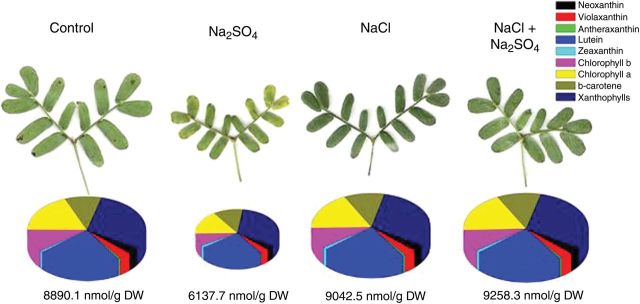
Distribution patterns of photosynthetic pigments in leaves of control and salt-treated *P. strombulifera* plants. Control plants (48 days); Na_2_SO_4_, NaCl and two-salt treated plants (*Ψ*_o_ = −2.6 MPa, 48 days). Area of circle corresponds to total content of pigments. Area of circle in leaves of NaCl + Na_2_SO_4_-treated plants corresponds to 100 %. Areas of circles (and portions thereof) for other treatments are proportional.

Levels of pigments in control and salt-treated plants at −2.6 MPa (48 days) are illustrated by pie charts in Fig. 2. At high salinity, only Na_2_SO_4_ treatment reduced the total pigment concentration, with evident chlorosis and leaf area reduction in these plants.

Chlorophyll *a* and *b* concentrations were not significantly affected by any salt treatment at −1.0 and −1.9 MPa (data not shown). However, both pigments showed a significant decrease in Na_2_SO_4_-treated plants at −2.6 MPa (−41 and −46 % for chlorophyll *a* and *b*, respectively, compared with controls). Similarly, total xanthophylls underwent a significant decrease in Na_2_SO_4_-treated plant leaves (−26 %). Total carotenoids, β-carotene, lutein, neoxanthin, violaxanthin and VAZ pigments (sum of violaxanthin (V), antheraxanthin (A) and zeaxanthin (Z)) concentrations showed a decrease under Na_2_SO_4_ treatment (Table 2), although only antheraxanthin diminution was statistically significant. Salt-induced variations in the content of the three single xanthophylls participating in the zeaxanthin cycle led to variations in the de-epoxidation (DEPS) index. The DEPS index showed the highest value in Na_2_SO_4_-treated leaves (42 % over control).

**Table 2.** Leaf pigment content (nmol g^−1^ DM) and pattern in *P. strombulifera* plants grown hydroponically at *Ψ*_o_ = −2.6 MPa, 48 days. Leaf pigment analysis was performed by HPLC-UV. Values represent the mean of three determinations. Values of the same line followed by different letters are significantly different (*P* = 0.05). DEPS index, de-epoxidation index; VAZ pigments, sum of violaxanthin, antheraxanthin and zeaxanthin.

	Control	Na_2_SO_4_	NaCl	NaCl + Na_2_SO_4_
	48 days		*Ψ*_o_ = −2.6 MPa	
Chlorophyll *a*	1669^b^	979^a^	1628^b^	1682^b^
Chlorophyll *b*	963^b^	516^a^	883^b^	910^b^
Total chlorophyll	2632^b^	1495^a^	2512^b^	2592^b^
Chl *a*/*b*	1.74^a^	1.9^a^	1.85^a^	1.85^a^
Lutein	1993^a^	1487^a^	2048^a^	2093^a^
Neoxanthin	271^a^	183^a^	272^a^	277^a^
Violaxanthin	307^a^	198^a^	301^a^	304^a^
Anteraxanthin	42^b^	17^a^	43^b^	39^b^
Zeaxanthin	71^a^	89^a^	105^a^	94^a^
VAZ	421^a^	304^a^	449^a^	437^a^
Xanthophylls	2684^b^	1973^a^	2768^b^	2808^b^
DEPS index	21.93^ab^	31.24^c^	27.86^bc^	25.96^bc^
β-Carotene	889^a^	699^a^	994^a^	1051^a^
Total carotenoids	3574^a^	2673^a^	3763^a^	3859^a^

### Polyphenols content under salt stress

**Figure 3.**
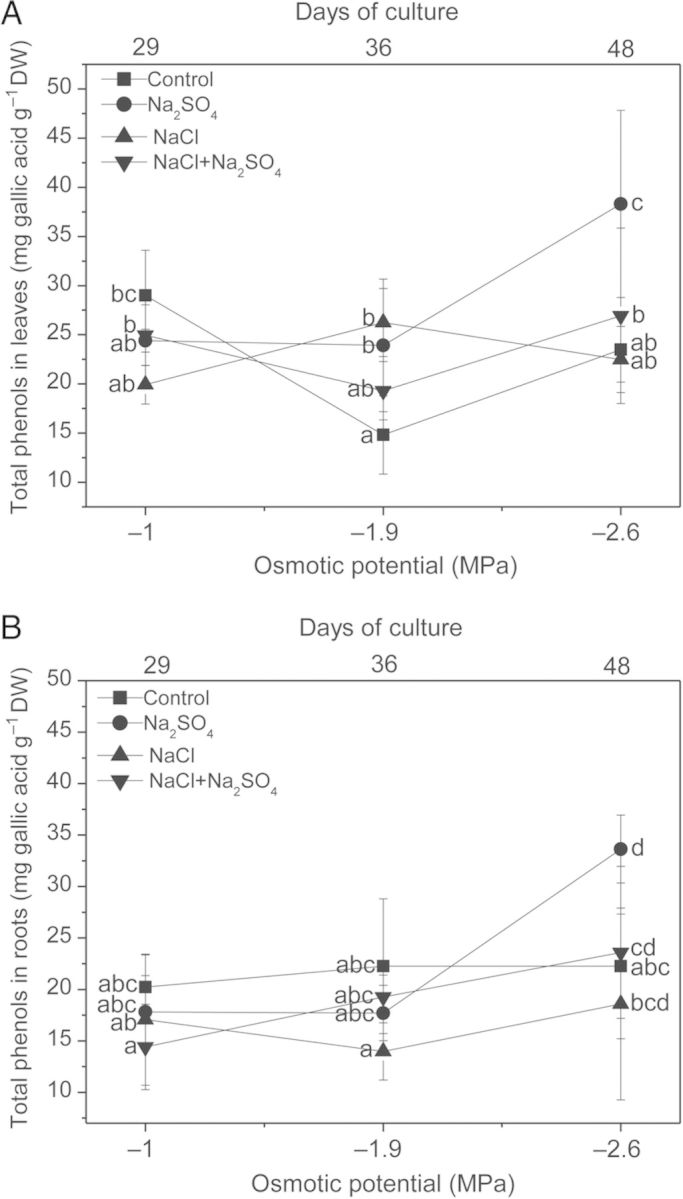
Effects of NaCl, Na_2_SO_4_ and their iso-osmotic mixture on total phenols content in leaves (A) and roots (B) of *P. strombulifera* plants. Salt treatments and osmotic potentials were compared by two-way ANOVA. Means values (±SE) followed by different letters are significantly different at *P* < 0.05 according to Bonferroni test (*n* = 6).

Total phenol concentration in leaves was significantly increased at moderate and high salinity (−1.9 and −2.6 MPa), principally in Na_2_SO_4_-treated plants (Fig. 3A). In roots, only Na_2_SO_4_ treatment at −2.6 MPa induced a significant increase in total phenols (Fig. 3B). In these plants it was mainly total flavonoids, flavan-3-ols and flavonols that significantly increased. Total flavonoids increased 45 % in leaves and 31 % in roots in relation to control plants; flavan-3-ols increased 40 % in leaves and 26 % in roots. Flavonols were significantly increased only in roots (Table 3).

**Table 3.** Polyphenol content (mg g^−1^ DM) in *P. strombulifera* plants grown hydroponically at *Ψ*_o_ = −2.6 MPa, 48 days. Total flavonoids, flavan-3-ols, condensed tannins, tartaric acid esters and flavonols were spectrophotometrically assayed. Values represent the mean of three determinations. Values of the same line followed by different letters are significantly different (*P* = 0.05).

	Control	Na_2_SO_4_	NaCl	NaCl + Na_2_SO_4_
	48 days		*Ψ*_o_ = −2.6 MPa	
Leaves				
Proanthocianidins	2.03648^a^	2.10446^a^	1.86098^a^	1.91553^a^
Flavan-3-ols	11.77364^a^	19.44889^b^	13.44172^a^	22.13718^b^
Flavonols	8.52674^a^	6.72473^a^	7.02322^a^	7.39824^a^
Tartaric acid esters	6.78241^a^	5.62903^a^	5.82525^a^	11.80177^a^
Total flavonoids	10.64836^a^	19.23884^b^	11.80177^a^	13.079^a^
Roots				
Proanthocianidins	2.13544^a^	2.29028^a^	1.61277^a^	2.21091^a^
Flavan-3-ols	12.11669^b^	16.14916^b^	2.97255^a^	8.11204^ab^
Flavonols	6.55984^a^	16.27267^b^	1.84187^a^	1.79613^a^
Tartaric acid esters	0.54348^a^	1.22436^a^	1.32964^a^	1.42049^a^
Total flavonoids	13.75591^ab^	19.72773^b^	10.50043^a^	11.63753^a^

**Figure 4.**
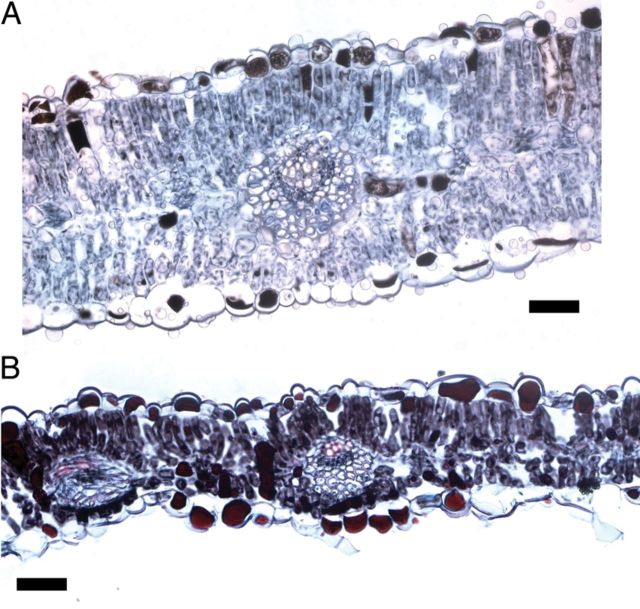
Cross-section of 48-day-old leaflets showing reduction in mesophyll thickness and polyphenols accumulation in cells of Na_2_SO_4_-treated plants. (A) Control; (B) Na_2_SO_4_-treated plants (*Ψ*_o_ = −2.6 MPa) (scale = 50 μm).

Leaflets of Na_2_SO_4_-treated plants presented great tannin accumulation in mesophyll and epidermal cells as well as thickness reduction (−30 % at high salinity) in relation to control plants as evidenced by the microscopic analysis (Fig. 4). This observation is in accordance with the polyphenol quantification described above. An important accumulation of tannins was also observed in other organs of Na_2_SO_4_-treated plants (roots, stems) (data not shown).

**Figure 5.**
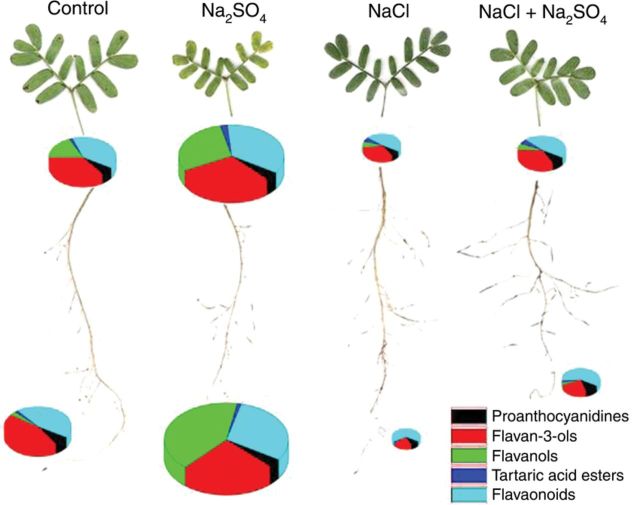
Distribution pattern of polyphenols in leaves and roots of control and salt-treated *P. strombulifera* plants. Control plants (48 days); Na_2_SO_4_, NaCl and two-salt treated plants (*Ψ*_o_ = −2.6 MPa, 48 days). Area of circle corresponds to total content of polyphenols in leaves (top) and roots (bottom). Area of circle in roots of Na_2_SO_4_-treated plants corresponds to 55.10 mg g^−1^ DW (100 %). Areas of circles (and portions thereof) for other plant organs and treatments are proportional.

Levels and pattern of different groups of polyphenols in control and salt-treated plants at −2.6 MPa (48 days) are illustrated by pie charts in Fig. 5. NaCl treatment did not affect polyphenol concentration in leaves; in roots, flavan-3-ols were significantly decreased (Table 3). On the contrary, Na_2_SO_4_ treatment sharply induced an increase in flavonoid compounds.

### Antioxidant activity of phenolic extracts

**Figure 6.**
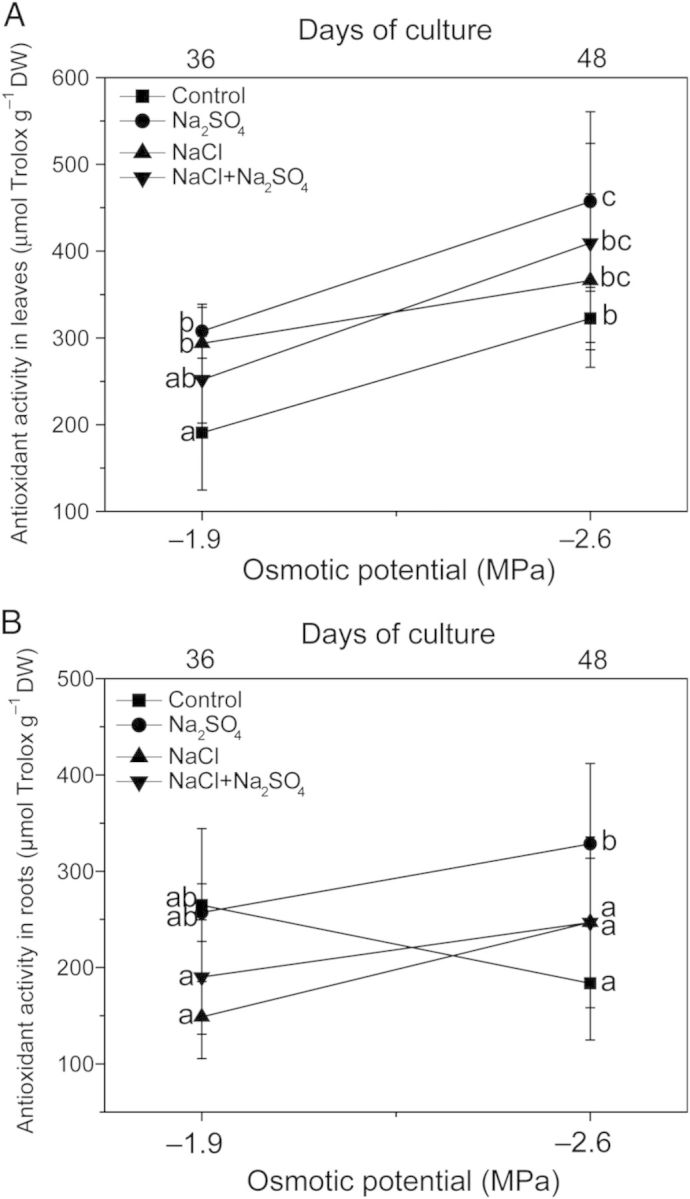
Effects of NaCl, Na_2_SO_4_ and their iso-osmotic mixture on antioxidant activity of phenolic extracts obtained from leaves (A) and roots (B) of *P. strombulifera* plants. Salt treatments and osmotic potentials were compared by two-way ANOVA. Means values (±SE) followed by different letters are significantly different at *P* < 0.05 according to Bonferroni test (*n* = 6).

Antioxidant activity was evaluated by testing the effect of polyphenolic extracts on the ABTS* radical. Antioxidant activity was directly correlated with total polyphenol concentration in leaves and roots. In leaves, an enhanced antioxidant activity was observed in Na_2_SO_4_ and NaCl-treated plants at −1.9 MPa. At −2.6 MPa only Na_2_SO_4_-treated plants presented a significant increase in antioxidant activity (25 % over control) (Fig. 6A). In roots, a significant increase in antioxidant activity was only observed in Na_2_SO_4_-treated plants at −2.6 MPa (45 % over control) (Fig. 6B).

## Discussion

Salt stress induces oxidative stress, as has been reported by numerous authors (Pang and Wang 2008; Abogadallah 2010; Tounekti *et al.* 2011), a component of which is caused by H_2_O_2_. Hydrogen peroxide is a versatile molecule that is involved in several plant cell processes under normal and stressful conditions. In the latter, H_2_O_2_ accumulates to high levels leading to an oxidative burst. However, increasing evidence indicates that H_2_O_2_ also functions as a signalling molecule in plants, acting as a secondary messenger (Quan *et al.* 2008). Recently, several authors have demonstrated that H_2_O_2_ accumulation under highly saline concentrations acts as a signal for an adaptative response to stress (Miller *et al.* 2010). Therefore, a tight control of H_2_O_2_ concentration is critical for cell homoeostasis.

In *P. strombulifera* plants, H_2_O_2_ concentration was significantly increased in roots of Na_2_SO_4_ and Na_2_SO_4_ + NaCl-treated plants at moderate salinity (−1.9 MPa) in correlation with increased lipid peroxidation in the former. Hernandez *et al.* (2010) observed that H_2_O_2_ was rapidly accumulated in *Brassica olareacea* roots during the first 24 h at 80 mM NaCl suggesting that this would be a signal for setting off the defence system in different organs of the plant. On the other hand, in the halophyte *Suaeda salsa* a significant decrease in H_2_O_2_ content was observed after 7-day NaCl treatment, indicating that this halophyte has an effective H_2_O_2_-scavenging system which is responsible for protection from the oxidative stress induced by salinity (Cai-Hong *et al.* 2005). This response would be quite general for halophytic species, as proposed by Bose *et al.* (2013). These authors argued that truly salt-tolerant species possessing efficient mechanisms for Na^+^ exclusion from the cytosol may not require a high level of antioxidant activity, as they simply do not allow excessive ROS production in the first instance. This would be the case of NaCl-treated plants of *P. strombulifera*, whose capacity for ion compartmentation and osmoregulation in their tissues and adequate hydric equilibrium has been demonstrated in our previous work (Llanes *et al.* 2013; Reginato *et al.* 2014).

In agreement with our previous results (Reginato *et al.* 2014), *P. strombulifera* showed a greater sensitivity to Na_2_SO_4_ than to NaCl, as indicated by the important oxidative damage induced in tissues when the SO_4_^2−^ anion is present in the medium. Malondialdehyde is considered a reliable indicator of oxidative stress resulting from degradation of membrane lipids under several abiotic constraints (Zhang and Kirkham 1996; Hernández *et al.* 2001). Thus, the significant increase in MDA concentration under Na_2_SO_4_ treatment correlates with the growth inhibition and metabolic disorder induced by this salt as previously reported (Llanes *et al.* 2013). It is worth noting that when SO_4_^2−^ and Cl^−^ are both present in the medium, ionic interactions between the two anions at the membrane level cause a partial reversion of the oxidative damage caused by SO_4_^2−^ in roots. This response is in agreement with the observation that NaCl + Na_2_SO_4_-treated plants showed intermediate values in growth parameters, compatible solute synthesis and ion content between those obtained with monosaline treatments, as previously demonstrated (Llanes *et al.* 2013; Reginato *et al.* 2014).

The lower MDA concentration in NaCl-treated plants suggests that this salt did not trigger a damaging oxidative burst as the one triggered by Na_2_SO_4_. Alternatively, this response maybe associated with the beneficial effects of putrescine accumulation in NaCl-treated plants (Reginato *et al.* 2012) which has been proposed as an inductor of the antioxidative defence system (Verma and Mishra 2005).

Salt tolerance in plants is also related to their aptitude to maintain their chlorophyll level, an efficient CO_2_ assimilation rate and stomatal conductance under salinity conditions (Redondo-Gómez *et al.* 2007). The chlorophyll response to salinity seems to depend on stress severity. Low salinities generally lead to an increase in chlorophyll levels whereas severe salinities often cause reduction (Wei *et al.* 2006). In some halophytes, photosynthesis has been shown to be unaffected by salinity, or even stimulated at low salt concentrations (Parida *et al.* 2004; Rabhi *et al.* 2011).

In the present experiments, *P. strombulifera* showed a good ability to tolerate elevated NaCl concentrations with chlorophyll concentration remaining unchanged with respect to controls, while Na_2_SO_4_ stress significantly reduced chlorophyll concentration. Similar to the results reported by Ramani *et al.* (2006), no changes in carotenoid concentrations were observed. In addition to their role as secondary light-absorbing pigments, carotenoids, and β-carotene in particular, are able to reduce the Chl triplet state and to prevent the formation of the harmful singlet oxygen or to scavenge it after its production by the interaction of triplet chlorophyll with O_2_ (Ramani *et al.* 2006). The unchanged carotenoid concentration, despite reduction in chlorophyll concentration, observed in Na_2_SO_4_-grown plants resulting in an increased carotenoid/chlorophyll ratio may represent a strategy to protect photosystems against photooxidation. One of the most effective mechanisms of excess energy dissipation is the de-epoxidation of violaxanthin to antheraxanthin and zeaxanthin through the xanthophyll cycle (VAZ) (Demmig-Adams and Adams 1992). Such a protective mechanism seems to be carried out by salinized plants of *P. strombulifera*, principally those grown in the presence of Na_2_SO_4_ with the maximal DEPS index, indicating the need to alleviate excessive excitation pressure. Concomitantly, these plants showed a remarkable decrease in the maximal photochemical efficiency (*F*_v_/*F*_m_) and electron transport rate at the end of the experiment (unpubl. res.). If there were inactive units in photosytem II, there would be great potential for ROS formation.

Plants vary widely in their phenolic content and composition, with both genetics and environment affecting the type and level of these compounds (Awika and Rooney 2004; De Abreu and Mazzafera 2005). Phenolic compounds exhibit antioxidant activity in tissues exposed to a wide range of environmental stressors by inactivating lipid free radicals or preventing decomposition of hydroperoxides into free radicals (Pokorný 2001; Agati *et al.* 2002; Babu *et al.* 2003; Pearse *et al.* 2005). Generally the accumulation of phenolics is stimulated in response to biotic/abiotic stresses (Dixon and Paiva 1995; Naczk and Shahidi 2004). Increase in total polyphenol content in different tissues under increasing salinity has been reported in a number of plants (Agastian *et al.* 2000; Muthukumarasamy *et al.* 2000; Navarro *et al.* 2006). In the present study, NaCl treatment did not affect polyphenol synthesis in *P. strombulifera* plants, which was different from the response to Na_2_SO_4_ treatment, which induced a sharp increase in total phenols and flavonoid compounds and consequently, in the antioxidant activity in both leaves and roots. As evidenced by the microscopic analysis, leaflets of Na_2_SO_4_-treated plants showed a highly increased polyphenol accumulation in mesophyll and epidermal cells, in agreement with Tattini *et al.* (2005) who proposed that the main sites of flavonoid accumulation in plants (including glycosilated forms) are the mesophyll, epidermis and subepidermis of photosynthetic tissues. Taken together, these observations lead to the proposal of a fundamental role of polyphenols in the protection of the photosynthetic apparatus under severe stress. Furthermore, from our results it could be inferred that when other ROS-detoxifying systems such as the xanthophyll cycle fail or are not effective enough, as in the case of Na_2_SO_4_-treated plants, polyphenol production is increased as an alternative detoxifying system.

In *P. strombulifera* the pool of total phenols is composed mainly by flavan-3-ols, in leaves and roots. Hernández (2007) reported that the levels of flavan-3-ols increased significantly after a water deficit treatment in leaves of *Cistus clusii* in field conditions, and suggested that accumulation of flavan-3-ols and proanthocyanidins might protect leaves from excess of ROS. These authors reported that accumulation of monomeric flavan-3-ols preceded accumulation of proanthocyanidins (condensed tannins) and evidence was provided for *in vivo* oxidation of flavan-3-ols to their respective quinones, indicating their importance as antioxidants in plants. In *P. strombulifera* NaCl-treated plants, flavonoids and mainly flavan-3-ols did not increase as much as when the SO_4_^2−^ anion was present in the solution (single salt or mixed salts). It might be thought that under NaCl treatment a more efficient xanthophyll cycle would render unnecessary investment of resources in flavonoid synthesis since, despite their protective functions, stress-induced increase of secondary plant products like polyphenols is often counteracted by a corresponding decrease in biomass (Selmar and Kleinwächter 2013). Accordingly, in this work we observed that the large increase in total polyphenols found under Na_2_SO_4_ treatment was accompanied by strong growth inhibition, contrary to what happened under NaCl treatment (Reginato *et al.* 2014).

Finally, it should be stated that our observations do not underestimate the importance of the enzymatic and non-enzymatic antioxidants known to have a fundamental role in ROS scavenging, which are currently being analysed in our laboratory.

## Conclusions

As previously reported, ionic interactions between different types of salt in salinized soils modify the biochemical and morpho-physiological responses of *P. strombulifera* plants to salinity (Reinoso *et al.* 2004, 2005; Sosa *et al.* 2005; Reginato *et al.* 2012, 2013, 2014; Llanes *et al.* 2013, 2014).

The increase in H_2_O_2_ production and lipid peroxidation when SO_4_^2−^ anion is present in the growth solution indicates that this anion induced a strong oxidative stress in plants, different from that produced by NaCl treatment. Additionally, the significant accumulation of flavonoids in tissues under Na_2_SO_4_ treatment and their powerful antioxidant activity indicates a role for these compounds in counteracting the oxidative damage induced by severe salt stress, particularly, ionic stress.

## Sources of Funding

This study was supported with funds from CONICET PIP No. 5628, PICT-ANPCYT No. 02232/07, SECYT-Universidad Nacional de Río Cuarto, Argentina, to V.L., MINCyT-MAE to V.L. and A.R., and a postdoctoral fellowship from CONICET to M.R.

## Contributions by the Authors

Experiments with hydropony for plant cultures and salt treatments were carried out by M.A.R.; H_2_O_2_ and MDA quantiﬁcation were carried out by M.A.R. and A.F.; pigment analysis was conducted by A.C. and polyphenols and their antioxidant activity by M.A.R. and A.C. Statistical analyses were conducted by M.A.R. and A.C. S.C. and A.R. provided their facilities (labs and equipment) to perform the analysis on oxidative stress and polyphenols, respectively. V.L. is the Head of the Plant Physiology Lab where the project is currently being carried on. M.A.R. and V.L. wrote and edited the manuscript, with insightful comments by all co-authors.

## Conflicts of Interest Statement

None declared.
